# Multi-Scale Temporal Coordinate Attention Network with Peak-Aware Mechanism for Rolling Bearing Fault Diagnosis Under Low Signal-to-Noise Ratio Conditions

**DOI:** 10.3390/s26092904

**Published:** 2026-05-06

**Authors:** Xin Zhang, Xinming Liu, Fan Chen, Quanlong Li, Li Zhang, Jiahao Tian

**Affiliations:** 1 School of Resources and Materials, Northeastern University at Qinhuangdao, Qinhuangdao 066004, China; zhangxin@neuq.edu.cn (X.Z.); 17730544582@163.com (X.L.); chenfan@mails.neu.edu.cn (F.C.); liql0814@163.com (Q.L.); 2 School of Information, Liaoning University, Shenyang 110036, China; zhang_li@lnu.edu.cn

**Keywords:** bearing fault diagnosis, deep learning, low signal-to-noise ratio, peak-aware attention mechanism, multi-scale temporal coordinate attention

## Abstract

Intelligent fault diagnosis of rolling bearings under high-noise industrial conditions remains a significant challenge. Traditional attention-based deep learning models often rely on global average pooling, which may inadvertently smooth out high-frequency transient impulses essential for fault identification, potentially leading to degraded performance in low signal-to-noise ratio (SNR) environments. To address this, we propose a Multi-Scale Temporal Coordinate Attention Network (MS-TCANet). The framework introduces a Peak-Aware Coordinate Attention (PACA) mechanism that combines max-pooling and average-pooling along directional coordinates. This dual-pooling design aims to better preserve transient impact features while maintaining a stable global representation, thereby mitigating the feature over-smoothing issue common in conventional attention modules. Additionally, an asymmetric multi-scale convolution block is incorporated to capture both short-term impacts and long-range periodic signatures. Experiments on three benchmark datasets (CWRU, Paderborn University, and XJTU-SY) indicate that the proposed MS-TCANet achieves favorable diagnostic accuracy compared to several representative and advanced methods, particularly under severe noise conditions (e.g., −10 dB SNR). t-SNE and Grad-CAM visualizations further suggest that the model can capture fault-related signatures more reliably than standard architectures in noisy environments.

## 1. Introduction

Rolling bearings are quintessential components in modern rotating machinery, acting as the fundamental joints in wind turbines, aerospace engines, high-speed trains, and manufacturing robotics [1,2]. Operating under prolonged heavy loads and harsh environments, bearings are highly susceptible to localized damages such as fatigue spalling and pitting [3]. The unexpected failure of these components can precipitate severe equipment breakdowns, critical safety incidents, and massive economic losses [4,5]. Therefore, accurate and timely fault diagnosis of rolling bearings is of paramount importance for ensuring industrial reliability and realizing intelligent condition-based maintenance (CBM) [6].

In recent years, data-driven approaches based on machine learning and deep learning (DL) have achieved remarkable success in intelligent equipment fault diagnosis, gradually replacing traditional signal processing paradigms that heavily rely on complex manual feature engineering and domain-expert knowledge [7,8]. The superiority of these advanced machine learning algorithms in handling complex industrial data has been extensively validated in recent top-tier studies [9,10]. Among various DL architectures, Convolutional Neural Networks (CNNs) have emerged as the mainstream paradigm due to their powerful end-to-end local representation learning capabilities from raw 1D vibration signals or 2D time-frequency images [11,12]. To capture more discriminative representations, advanced structural variants have been extensively explored. For instance, Deep Residual Networks (ResNet) [13] were introduced into mechanical diagnosis to alleviate the vanishing gradient problem in extremely deep architectures [14]. Furthermore, Deep Residual Shrinkage Networks (DRSN) elegantly integrated channel-wise soft thresholding mechanisms to eliminate irrelevant noise features [15,16], establishing a robust baseline for vibration signal processing [17].

Despite these tremendous advancements, performing reliable diagnosis in real-world industrial scenarios remains a formidable challenge [12,18]. Recently, several pioneering works have explicitly highlighted the critical bottleneck of extreme industrial noise and variable operating conditions. For instance, Zou et al. [19] emphasized the severe degradation of diagnostic reliability in marine gearboxes under extreme environmental noise, proposing a hybrid denoising network. Similarly, Shi et al. [20] investigated the rigorous extraction of weak periodic characteristic information deeply submerged in strong background noise. Furthermore, the combined threats of environmental interference and time-varying operating conditions in wind turbines were meticulously addressed by Wei and Yuan [21]. The vibration signals collected by industrial sensors are inherently contaminated by heavy background noise generated by adjacent mechanical components, varying operational loads, and complex transmission paths [8,22]. In such environments, the weak transient impulses caused by incipient localized defects are often entirely submerged in the background interference [17]. Consequently, the signal-to-noise ratio (SNR) of the acquired data can plunge to 0 dB or even −10 dB. Under these harsh conditions, the diagnostic performance and feature boundaries of conventional CNNs and standard ResNets degrade drastically, severely hindering their practical deployment [23,24].

To enhance noise robustness and feature sensitivity, attention mechanisms have been widely adopted in fault diagnosis to force networks to dynamically focus on informative fault signatures while suppressing ambient background noise [25]. Mainstream attention modules have evolved rapidly, ranging from the pioneering Squeeze-and-Excitation (SE) networks [26] and Convolutional Block Attention Module (CBAM) [27], to more efficient architectures like Efficient Channel Attention (ECA) [28] and Coordinate Attention (CoordAtt) [29].

To address these critical challenges, recent studies have explored attention-guided and multi-scale feature enhancement paradigms. For instance, hybrid models incorporating variational mode decomposition and attention-weighted networks have been utilized to suppress noise interference [30]. Furthermore, adaptive 1D multi-channel convolutional networks leveraging attention mechanisms and sparse structures have been proposed to boost diagnostic capabilities in noisy environments [31,32]. Along similar multi-scale perspectives, He et al. [33] proved that employing multi-scale dilated convolutions drastically mitigates the effect of background noise while capturing informative diagnostic features. Furthermore, recent advancements have demonstrated the remarkable efficacy of embedding physical information into multi-scale deep learning structures, such as utilizing multi-scale physical neural networks for robust multiaxial fatigue life prediction [34].

However, a critical limitation persists from the perspective of physical signal characteristics. Conventional attention modules uniformly employ Global Average Pooling (GAP) to aggregate spatial feature maps into global descriptors [35]. While average pooling acts as an effective low-pass filter to smooth Gaussian white noise, it fatally dilutes the sharp, high-frequency peak features that physically characterize the transient fault impulses of bearings [36]. When the background noise is overwhelming, this global over-smoothing effect strips away the most critical diagnostic evidence, inevitably leading to substantial mode collapse and misclassification. Additionally, modern large-kernel designs (e.g., ConvNeXt [37] and RepLKNet [38]) have proven that asymmetric and expanded receptive fields are vital for capturing long-range periodic modulations, yet they are rarely customized for the dual temporal-frequency nature of mechanical signals [36].

To overcome the aforementioned bottlenecks, this paper proposes a novel robust framework named Multi-Scale Temporal Coordinate Attention Network (MS-TCANet). The core motivation is to rigorously bridge the gap between deep AI architecture design and the intrinsic physical characteristics of noisy vibration signals. Specifically, we design a Peak-Aware Coordinate Attention (PACA) mechanism that introduces a parallel max-pooling branch alongside the traditional average-pooling pathway. This dual-pooling design enables the network to actively capture and preserve high-frequency impulse peaks (via max-pooling) while simultaneously stabilizing the global operational state representation (via average-pooling). Furthermore, recognizing that bearing signals concurrently contain short-duration impacts and long-duration modulations, a robust multi-scale module utilizing asymmetric large-kernel convolutions (3×3 and 7×7) is constructed to extract multi-resolution transient and periodic features within a single layer.

The main contributions of this paper are summarized as follows:

- An MS-TCANet framework is developed for end-to-end bearing fault diagnosis, which aims to improve robustness and feature extraction capabilities under high-noise industrial conditions, including extreme scenarios down to −10 dB SNR.
- A Peak-Aware Coordinate Attention (PACA) mechanism is designed to address the feature over-smoothing issue common in standard attention methods. By combining average and max pooling along directional coordinates, the mechanism helps preserve weak transient fault impulses that are often obscured by heavy background noise.
- A multi-scale large-kernel convolution block is integrated into the architecture, enabling the network to capture both localized high-frequency impacts and long-term periodic degradation signatures within a unified layer.
- Comparative experiments and ablation studies are conducted on three benchmark datasets (CWRU, Paderborn University, and XJTU-SY). The results, supported by t-SNE and Grad-CAM visualizations, indicate that the proposed MS-TCANet achieves favorable diagnostic performance compared to several advanced and representative methods in noisy acoustic environments.

## 2. Theoretical Background

### 2.1. Standard Coordinate Attention

Coordinate Attention (CoordAtt) is highly regarded for its ability to embed positional information into channel attention, which effectively enhances the representational power of standard Convolutional Neural Networks (CNNs). Unlike Squeeze-and-Excitation (SE) networks that employ 2D global average pooling (GAP) to compress spatial information into a single channel descriptor, standard CoordAtt factorizes the 2D spatial pooling into two 1D feature encoding operations along the horizontal and vertical directions.

Given an input feature map $X\in R^{C\times H\times W}$, the standard CoordAtt extracts the intermediate representation along the horizontal direction *h* and vertical direction *w* using 1D average pooling. The pooled output for the *c*-th channel at height *h* and width *w* can be formulated as Equations (1) and (2):

(1) $$z_{c,avg}^{h}\left(h\right)=\frac{1}{W}\sum_{i=1}^{W}X_{c}\left(h,i\right)$$

(2) $$z_{c,avg}^{w}\left(w\right)=\frac{1}{H}\sum_{j=1}^{H}X_{c}\left(j,w\right)$$

Subsequently, the aggregated feature maps are concatenated and passed through a shared 1×1 convolutional transformation function to generate intermediate spatial awareness maps. Finally, the maps are split and activated via a sigmoid function to produce the attention weights in both spatial directions.

However, under extremely noisy conditions (e.g., SNR ≤0 dB), the background noise energy completely overwhelms the diagnostic signal. The standard average pooling operations described in Equations (1) and (2) act strictly as low-pass filters. While they suppress high-frequency white noise, they inevitably smooth out and obliterate the critical high-frequency transient impulses generated by early bearing faults, leading to a drastic decline in diagnostic accuracy.

### 2.2. Proposed MS-TCANet Framework

To address the feature over-smoothing dilemma and effectively extract fault signatures under extreme industrial noise, this section details the proposed Multi-Scale Temporal Coordinate Attention Network (MS-TCANet).

#### 2.2.1. Overall Architecture

The raw 1D vibration signal is inherently non-stationary. Thus, we first employ the Short-Time Fourier Transform (STFT) to map the noisy 1D time-series into a 2D time-frequency representation, which simultaneously retains temporal transients and frequency band distributions. The 2D STFT magnitude spectrogram is then normalized and fed into the MS-TCANet.

**Figure 1:**
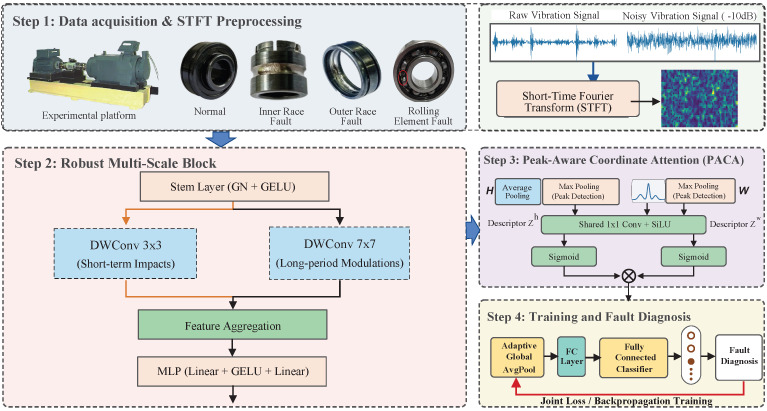
The overall framework of the proposed MS-TCANet for bearing fault diagnosis. The solid arrows indicate the direction of data flow.

As illustrated in Figure 1, the overarching architecture of MS-TCANet avoids traditional Batch Normalization (BN). Instead, it adopts Group Normalization (GN) and Layer Normalization (LN). This deliberate design is inspired by the fact that under severe noise, the batch-wise statistical properties shift unpredictably. By normalizing across the channel dimension independently for each sample, the network’s resilience against domain-shift caused by heavy noise is fundamentally strengthened. The network consists of a robust convolutional stem, followed by three hierarchical feature extraction stages. Each stage is constructed by stacking the proposed Robust Multi-Scale Blocks and down-sampling layers. Finally, an adaptive average pooling layer and a fully connected classifier are utilized for fault diagnosis. To ensure reproducibility, the detailed architectural parameters are specified as follows. The initial stem utilizes a 4×4 convolution with a stride of 2 and padding of 1 to map the 1-channel input to 64 channels, followed by GN and GELU. The Group Normalization (GN) in our framework is consistently configured with num_groups = 1, making it operate as a sample-wise Layer Normalization. The network hierarchically cascades three stages with channel dimensions of [64, 128, 256]. Each stage is composed of exactly 2 Robust Multi-Scale Blocks. Spatial downsampling between adjacent stages is implemented using a separate layer comprising GN followed by a non-overlapping 2×2 convolution with a stride of 2.

#### 2.2.2. Multi-Scale Large-Kernel Convolution Block

Bearing vibration signals inherently contain both short-duration high-frequency impacts (e.g., a rolling element hitting a localized spall) and long-duration low-frequency structural modulations (e.g., shaft rotational speed). A single uniform convolution kernel is insufficient to capture this diversity.

Therefore, we construct a Multi-Scale Block utilizing depthwise separable convolutions with asymmetric receptive fields. Given the input feature $X_{in}$, the multi-scale aggregation is mathematically defined as in Equation (3):

(3) $$X_{ms}=\frac{1}{2}\left({\mathrm{DWConv}}_{3\times 3}\left(X_{in}\right)+{\mathrm{DWConv}}_{7\times 7}\left(X_{in}\right)\right)$$

where ${\mathrm{DWConv}}_{3\times 3}$ captures the localized fine-grained impact characteristics, and the large-kernel ${\mathrm{DWConv}}_{7\times 7}$ provides a broader receptive field to encompass periodic fault modulations. The aggregated feature $X_{ms}$ is then processed by a pointwise Multi-Layer Perceptron (MLP) consisting of Layer Normalization, a channel-expansion linear layer (4× ratio), a GELU activation, and a channel-reduction linear layer.

#### 2.2.3. Peak-Aware Coordinate Attention (PACA) Mechanism

The core innovation of the MS-TCANet lies in the proposed Peak-Aware Coordinate Attention (PACA) module, which replaces the vulnerable standard CoordAtt. The fundamental motivation of PACA is to introduce a “peak-sensitive” radar to the attention mechanism, counteracting the excessive smoothing effect of average pooling.

In PACA, we establish a dual-pooling paradigm. Alongside the 1D average pooling branch, a parallel 1D max pooling branch is introduced. Max pooling inherently acts as a peak detector, capturing the maximum amplitude responses corresponding to transient fault impulses. The horizontal and vertical spatial encoding processes in PACA are formulated as Equations (4) and (5):

(4) $$z_{c}^{h}\left(h\right)=\underset{\mathrm{Average}\;\mathrm{Pooling}}{\underset{︸}{\frac{1}{W}\sum_{i=1}^{W}X_{c}\left(h,i\right)}}+\underset{\mathrm{Max}\;\mathrm{Pooling}}{\underset{︸}{\max_{1\leq i\leq W}X_{c}\left(h,i\right)}}$$

(5) $$z_{c}^{w}\left(w\right)=\underset{\mathrm{Average}\;\mathrm{Pooling}}{\underset{︸}{\frac{1}{H}\sum_{j=1}^{H}X_{c}\left(j,w\right)}}+\underset{\mathrm{Max}\;\mathrm{Pooling}}{\underset{︸}{\max_{1\leq j\leq H}X_{c}\left(j,w\right)}}$$

While dual-pooling operations have been utilized in classic attention modules (e.g., CBAM), they typically apply pooling globally across spatial dimensions, which inevitably discards the precise positional coordinates of the features. In contrast, PACA performs directional 1D pooling, which preserves the exact frequency and temporal coordinates necessary for locating transient impacts in the time-frequency domain.

By directly summing the average-pooled and max-pooled representations, the formed intermediate tensors $z^{h}\in R^{C\times H\times 1}$ and $z^{w}\in R^{C\times 1\times W}$ concurrently encapsulate the global background noise distribution and the sharp diagnostic peaks. Because the input 2D STFT spectrograms are initially standardized and the intermediate features are regulated by Group Normalization, the average and max-pooled tensors reside in comparable numerical scales. Consequently, direct element-wise addition is employed to fuse the branches. This avoids introducing extra parameters, while the subsequent 1×1 convolution ($F_{1}$) serves as a learnable linear transformation to balance the aggregated features.

Since these two direction-aware feature tensors possess mismatched spatial dimensions, $z^{w}$ is first transposed to ${\left(z^{w}\right)}^{T}\in R^{C\times W\times 1}$. They are then concatenated along the spatial dimension and mapped to a lower-dimensional space using a shared 1×1 convolutional layer $F_{1}$, followed by a SiLU activation, as defined in Equation (6):

(6) $$f=\delta \left(\mathrm{GN}\left(F_{1}\left(\left[z^{h},{\left(z^{w}\right)}^{T}\right]\right)\right)\right)$$

where [·,·] denotes the concatenation operation, GN(·) is Group Normalization, δ(·) represents the SiLU activation, and $f\in R^{C/r\times (H+W)\times 1}$ is the fused intermediate feature map. The reduction ratio is set to r=32, with a minimum channel limit enforced (e.g., 8 channels) to preserve representational capacity.

Subsequently, the tensor f is split along the spatial dimension into two separate tensors: $f^{h}\in R^{C/r\times H\times 1}$ and $f^{w\_temp}\in R^{C/r\times W\times 1}$. The latter is then transposed back to its original spatial orientation, yielding $f^{w}\in R^{C/r\times 1\times W}$. Two additional 1×1 convolutions ($F_{h}$ and $F_{w}$) are utilized to up-sample the channel dimensions back to the original size *C*. The attention weight maps $a^{h}$ and $a^{w}$ are obtained via the sigmoid function σ, as shown in Equation (7):

(7) $$a^{h}=\sigma \left(F_{h}\left(f^{h}\right)\right),\;a^{w}=\sigma \left(F_{w}\left(f^{w}\right)\right)$$

The attended feature map is computed by applying the generated weights to the original input feature *X* via element-wise multiplication. Finally, a residual connection is employed to yield the ultimate output of the block $Y\in R^{C\times H\times W}$, calculated by Equation (8):

(8) $$Y_{c}\left(h,w\right)=X_{c}\left(h,w\right)+\left(X_{c}\left(h,w\right)\times a_{c}^{h}\left(h\right)\times a_{c}^{w}\left(w\right)\right)$$

By dynamically attending to both positional dependencies and transient impact peaks, the PACA module empowers the MS-TCANet to maintain high fault-feature sensitivity in −10 dB environments. The comprehensive architecture, coupled with multi-scale large kernels and residual learning, effectively prevents the network from collapsing under heavy noise interference.

## 3. Implementation Details

All experiments were accelerated by a workstation equipped with an Intel Core i7-9750H CPU @ 2.60 GHz, 16.0 GB of RAM, and an NVIDIA GeForce RTX 2060 GPU. The deep learning models were implemented using the PyTorch framework in a Python 3.9.24 environment, supported by CUDA version 13.0 for efficient GPU parallel computing.

For each dataset, the raw 1D vibration signals were segmented into non-overlapping sliding windows of 1024 data points. These segmented samples were then randomly divided into training, validation, and testing sets with a ratio of 70%, 10%, and 20%, respectively. The Short-Time Fourier Transform (STFT) was applied to convert these segments into normalized 2D time-frequency spectrograms, serving as the network inputs.

It should be clarified that real industrial signals are rarely corrupted by pure white noise; they typically suffer from non-Gaussian interferences, such as spikes and transient fluctuations correlated with the pulse-width modulation (PWM) of inverter drives. In this study, Additive White Gaussian Noise (AWGN) was dynamically injected into the raw vibration signals primarily as a standard benchmark to stress-test the noise-resistance boundary of the models. The Signal-to-Noise Ratio (SNR) was configured from 10 dB down to an extreme −10 dB. It is worth noting that, although the models were quantitatively evaluated under various noise conditions (e.g., strong noise at −4 dB and severe noise at −10 dB, as shown in the subsequent metric tables), the qualitative visual analyses (i.e., confusion matrices, attention heatmaps, and t-SNE manifolds) in Section 4 strictly focus on the severe −10 dB scenario. This “worst-case” analytical strategy is deliberately adopted to explicitly expose the feature extraction boundaries and the ultimate robustness of the comparative models.

During the optimization process, the models were trained using the AdamW optimizer with a learning rate of 1 × 10^−3^ and a weight decay of 1 × 10^−4^. A Cosine Annealing scheduler was employed to smoothly decay the learning rate. The batch size was set to 64, and the network was optimized over 100 training epochs.

To ensure the statistical reliability of the diagnostic results and eliminate the influence of accidental randomness, all experiments in this study were repeated 10 times using different random seeds. The final diagnostic performance is reported as the “Mean ± Standard Deviation”. This rigorous evaluation protocol guarantees that the observed superiority of MS-TCANet under severe noise is consistent and reproducible.

### 3.1. Comparison Methods

To comprehensively verify the superiority and noise robustness of the proposed MS-TCANet, four representative state-of-the-art deep learning models are selected as baseline comparison methods. These models cover classic architectures, attention mechanisms, noise-reduction specific networks, and modern large-kernel designs:

- ResNet [13]: A classic Deep Residual Network (ResNet-18 is utilized in this paper). It serves as the fundamental baseline to evaluate the basic feature extraction capability of standard deep architectures without specific noise-aware modules.
- ECA-CNN [28]: A CNN architecture integrated with the Efficient Channel Attention (ECA) module. It represents the mainstream attention-guided models, aiming to verify the performance difference between traditional global average pooling (GAP) based attention and our proposed PACA mechanism.
- DRSN [15]: Deep Residual Shrinkage Network, a highly recognized fault diagnosis model specifically designed for noisy environments. It employs a soft-thresholding sub-network to eliminate noise-related features, serving as a powerful state-of-the-art competitor in anti-noise tasks.
- ConvNeXt [37]: A modernized pure CNN architecture that incorporates large-kernel designs (e.g., 7×7) and advanced macro-designs. It is selected to validate whether our customized asymmetric multi-scale large-kernel block is more suitable for 1D/2D mechanical vibration signals than universal computer vision large-kernel models.

For a fair and rigorous comparison, all the baseline models are reproduced under the exact same experimental setups, including identical dataset partitioning, data preprocessing strategies, loss functions, and optimization hyperparameters.

### 3.2. Dataset Descriptions

To avoid the “dataset bias” commonly found in single-dataset evaluations, three highly respected bearing fault datasets with varying degrees of diagnostic difficulty were utilized: Case Western Reserve University (CWRU), Paderborn University (PU), and Xi’an Jiaotong University and Changxing Sumian Yaguo (XJTU-SY).

#### 3.2.1. Case Western Reserve University (CWRU) Dataset

**Figure 2:**
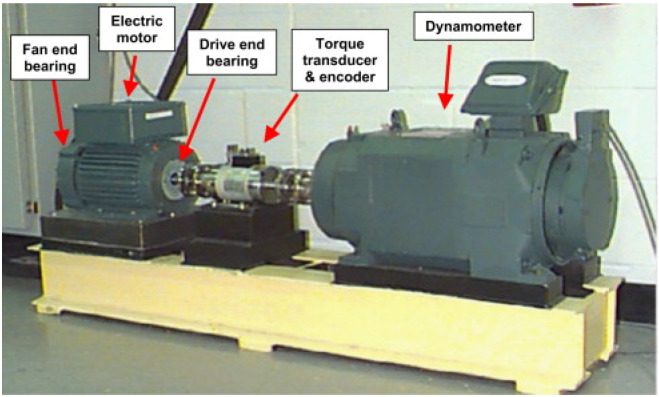
The experimental testbed of the CWRU bearing dataset, including a motor, a torque transducer, and a dynamometer.

The CWRU dataset [39] is widely recognized as a standard benchmark for bearing fault diagnosis. As illustrated in Figure 2, the experimental testbed primarily consists of a 2 horsepower (hp) electric motor, a torque transducer/encoder, and a dynamometer.

Single-point faults were artificially introduced into the test bearings (SKF deep groove ball bearings) using electro-discharge machining (EDM). The vibration signals were collected by an accelerometer mounted on the drive end of the motor housing at a sampling frequency of 12 kHz.

To simulate different fault severities and locations, the dataset includes three types of defects: Rolling Element (Ball) defect, Inner Race (IR) defect, and Outer Race (OR) defect. Each defect type features three different damage diameters: 0.007 inches, 0.014 inches, and 0.021 inches. Along with the normal operating state, a total of 10 distinct health conditions are constructed for the classification task. The detailed description of the 10 health states under a typical motor load of 1 hp is summarized in Table 1.

#### 3.2.2. Paderborn University (PU) Dataset

To evaluate the models’ feature extraction boundaries under more complex and realistic industrial conditions, the Paderborn University (PU) bearing dataset [40] is adopted as the second benchmark. Unlike the CWRU dataset, which strictly utilizes artificial defects, the PU dataset incorporates both artificially induced damages and real physical damages (such as pitting and fatigue) caused by accelerated life tests. These natural wear-and-tear characteristics generate much weaker and more complex fault impulses, making the diagnostic task significantly more challenging.

**Figure 3:**
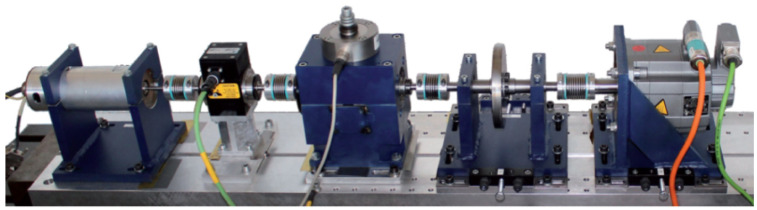
The modular experimental test rig for the PU bearing dataset, comprising a drive motor, test module, and load module.

As shown in Figure 3, the modular test rig consists of several main components: an electric drive motor, a torque-measurement shaft, a rolling bearing test module, and a load module. Vibration signals were acquired by a piezoelectric accelerometer mounted on the test module housing at a high sampling rate of 64 kHz.

For a consistent cross-dataset evaluation, the vibration data from the PU dataset were downsampled and uniformly partitioned into segments of 1024 data points. We selected data collected under the standard operating condition (rotational speed of 1500 rpm, load torque of 0.1 Nm, and radial force of 1000 N). To align with the unified evaluation framework, the selected bearings with varying damage degrees and locations are mapped into 10 standard health states, utilizing a consistent nomenclature (Severity 1 to 3 corresponding to labels like _007, _014, and _021). The detailed categorization is provided in Table 2.

#### 3.2.3. XJTU-SY Bearing Dataset

To further validate the dynamic feature tracking capability and robustness of the proposed MS-TCANet against time-varying operating conditions, we utilize the XJTU-SY bearing dataset [41] as our final benchmark. Unlike the static fault scenarios in the CWRU and PU datasets, the XJTU-SY dataset captures the full run-to-failure (RTF) accelerated degradation process of bearings. The fault signatures in this dataset naturally evolve from incipient anomalies to severe breakdowns, presenting highly non-stationary distribution shifts.

**Figure 4:**
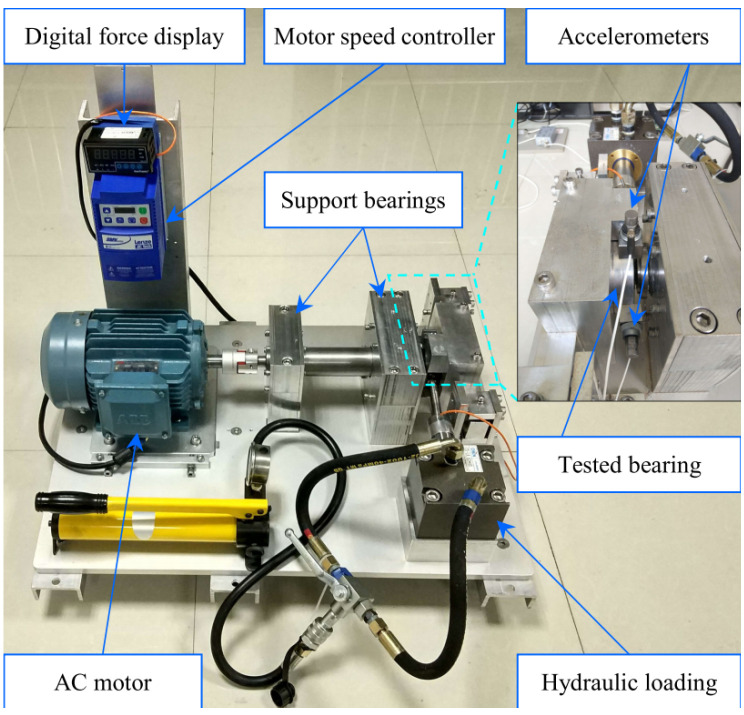
The run-to-failure experimental testbed of the XJTU-SY bearing dataset, featuring a hydraulic loading system and an AC induction motor.

The experimental testbed, as illustrated in Figure 4, consists of an alternating current (AC) induction motor, a motor speed controller, a support shaft, two support bearings, a hydraulic loading system, and the test bearing (LDK UER204). The vibration signals were captured by two PCB 352C33 accelerometers placed on the housing of the test bearing on the horizontal and vertical axes, with a sampling frequency of 25.6 kHz.

For our experiments, horizontal vibration signals from bearings operating under various working conditions (e.g., 35 Hz/12 kN, 37.5 Hz/11 kN) were utilized. To maintain a rigorous and unified evaluation framework across all three datasets, we adopted a degradation-stage mapping strategy. Specifically, the continuous run-to-failure lifecycle of the selected bearings with inner race, outer race, and rolling element faults was segmented into early, middle, and late degradation stages based on the amplitude of time-domain indicators. These natural degradation stages are systematically mapped to the equivalent unified class labels (Severity 1 to 3, denoted as _007, _014, and _021, respectively). The resulting 10 health states are outlined in Table 3.

## 4. Experiments and Results

In this section, we comprehensively evaluate the proposed MS-TCANet on three representative bearing datasets: CWRU, PU, and XJTU-SY. To rigorously simulate real-world industrial environments, all experiments are conducted under varying Additive White Gaussian Noise (AWGN) levels, ranging from a benign 10 dB down to an extreme −10 dB.

It should be noted that the performance baseline differs across the three datasets due to their distinct physical characteristics. The CWRU dataset utilizes artificial EDM defects generating strong impact energies, whereas the PU dataset contains real physical fatigue damages with complex transmission paths, resulting in much weaker fault impulses. The XJTU-SY dataset further introduces non-stationary feature shifts by recording run-to-failure processes under varying loads. Consequently, the absolute diagnostic accuracy drops naturally on the PU and XJTU-SY datasets under −10 dB noise. However, evaluating across these varying difficulties helps verify the relative robustness of the models.

### 4.1. Performance Trend Analysis Across Varying SNRs

To systematically evaluate the diagnostic robustness and feature degradation boundaries of the models, we plot the comprehensive performance trend lines across the full SNR spectrum (from 10 dB down to −10 dB) for all three benchmark datasets.

**Figure 5:**
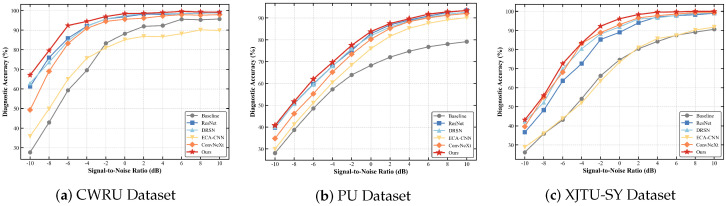
Diagnostic accuracy trends of different models across the full SNR spectrum.

As illustrated in Figure 5, in relatively benign acoustic environments (SNR ≥2 dB), all comparative models exhibit saturated and high diagnostic accuracy. This indicates that standard deep learning architectures possess sufficient capacity to extract discriminative fault features when the signal energy is dominant. However, as the background noise becomes catastrophic (SNR <0 dB), a universal performance degradation is observed. Standard architectures lacking deep feature refinement, such as the Baseline 2D-CNN and ECA-CNN, experience a drastic collapse, with their accuracy dropping most significantly.

While advanced deep residual networks (e.g., ResNet and DRSN) demonstrate strong baseline resistance to noise—maintaining a tight secondary cluster—they ultimately fail to break through the performance bottleneck under extreme masking. In sharp contrast, the proposed MS-TCANet (denoted by the thick red curve with star markers) consistently acts as the performance upper bound across the entire spectrum. Particularly in the most demanding −10 dB scenario, MS-TCANet persistently maintains the highest accuracy threshold across all three datasets. This full-range superiority explicitly confirms that the designed Peak-Aware Coordinate Attention mechanism effectively penetrates the noise floor to preserve critical physical fault signatures, guaranteeing highly reliable diagnostics even when competitor models saturate or collapse.

### 4.2. Evaluation on CWRU Dataset

The CWRU dataset is widely utilized to validate the basic diagnostic capability of models. In this section, we compare the proposed MS-TCANet with six state-of-the-art models. To highlight the robustness against non-stationary noise, we focus on the comparative metrics under strong (−4 dB) and severe noise (−10 dB) conditions, as detailed in Table 4.

The results in Table 4 indicate that MS-TCANet achieves superior diagnostic performance. In the −10 dB scenario, our model reaches an accuracy of 74.67%, outperforming the second-best model (DRSN) by a significant margin of 4.45%. This confirms the effectiveness of our multi-scale architecture in capturing robust features amidst high-level interference.

#### 4.2.1. Confusion Matrix Analysis

To intuitively evaluate the fine-grained classification capability and reveal the specific failure modes of different architectures, we visualize the confusion matrices of all comparative models on the CWRU dataset. We specifically focus on the severe noise scenario (SNR = −10 dB).

**Figure 6:**
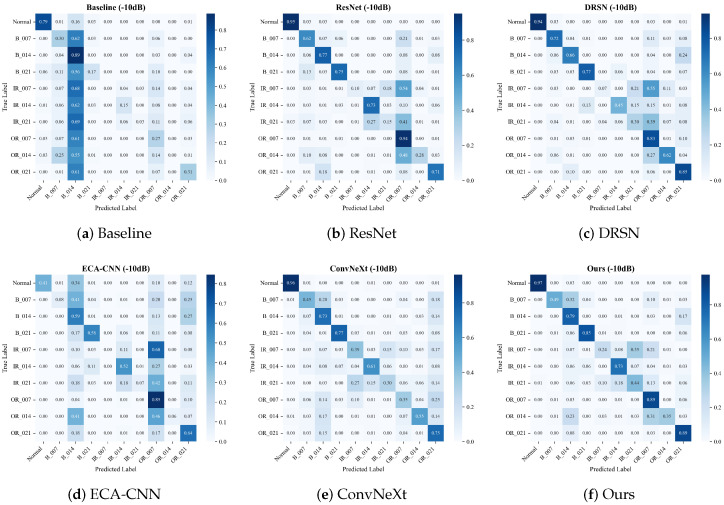
Confusion matrices on the CWRU dataset under Severe Noise (SNR = −10 dB).

When the noise further intensifies to −10 dB (where the noise energy is ten times that of the original signal), the diagnostic task reaches a physical bottleneck. As depicted in Figure 6, traditional models face drastic feature collapse.

The Baseline model exhibits a severe “mode collapse” phenomenon, overwhelmingly biasing its predictions toward the B_014 class and losing its discriminative boundaries. The ECA-CNN model also experiences unacceptable degradation, with its accuracy on the B_007 class dropping to a mere 8%, and even failing to reliably detect the Normal state (41%). Although deeper networks like ResNet and ConvNeXt resist complete collapse, their prediction confidence scatters widely across off-diagonal regions (e.g., ResNet yields only 28% on OR_014, and ConvNeXt drops to 30% on IR_021).

By contrast, the proposed MS-TCANet still maintains the clearest block-diagonal structure among all competitors. It successfully anchors the diagnostic boundaries for most classes, retaining an impressive 97% accuracy on Normal, 89% on OR_007, and 89% on OR_021. This visually proves that the proposed multi-scale receptive fields and peak-aware mechanisms act as a robust “safety net”, effectively penetrating the severe noise floor to capture intrinsic defect impulses and preventing substantial model breakdown.

#### 4.2.2. Attention Mechanism Visualization

To further demystify the internal decision-making mechanisms of the networks and verify whether the models genuinely capture the fault signatures rather than overfitting to background noise, we employ Grad-CAM to generate attention heatmaps. Following the “Strategy of the Hardest Cases”, we specifically visualize the most challenging B_007 (Rolling Element Defect) and IR_007 (Inner Race Defect) categories under the severe noise scenario (SNR = −10 dB).

**Figure 7:**
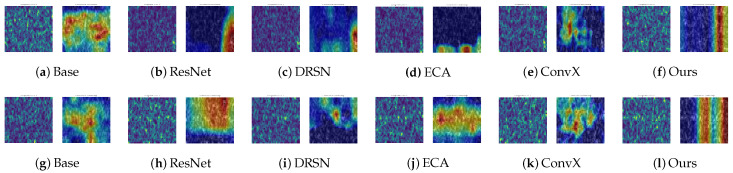
Grad-CAM attention heatmaps for B_007 (top) and IR_007 (bottom) on CWRU dataset at −10 dB SNR. The colors ranging from dark blue to dark red indicate the transition of attention weights from low to high.

Figure 7 presents a comprehensive visual comparison across all six models. The left column of each subfigure displays the original Short-Time Fourier Transform (STFT) spectrum heavily corrupted by −10 dB noise, while the right column superimposes the attention heatmap.

As observed, under such severe noise floors, the topological structure of the defect impulses is almost completely overwhelmed by background interference. Consequently, the Baseline model completely loses its focal points, exhibiting highly dispersed attention that erroneously highlights global noise patches. This visual evidence perfectly explains its severe mode collapse observed in the confusion matrix.

Other advanced architectures, despite their depth, also struggle to filter out the interference. For instance, the attention regions of ResNet and DRSN are highly fragmented. While advanced models like ECA-CNN and ConvNeXt attempt to gather features, their focal points remain misaligned and fail to accurately lock onto the periodic impact signatures of the bearings.

In sharp contrast, the proposed MS-TCANet (Ours) exhibits an exceptional capability for noise-resistant feature localization. Its attention heatmaps demonstrate highly concentrated, distinct vertical bands. These bands perfectly align with the intrinsic periodic transient impulses of the bearing faults in the time-frequency domain. This visual evidence convincingly corroborates that the proposed multi-scale receptive fields and peak-aware mechanisms can robustly penetrate the −10 dB noise floor, acting as a powerful “feature anchor” to maintain high diagnostic accuracy when other competitors fail.

#### 4.2.3. Feature Manifold Visualization (t-SNE)

**Figure 8:**
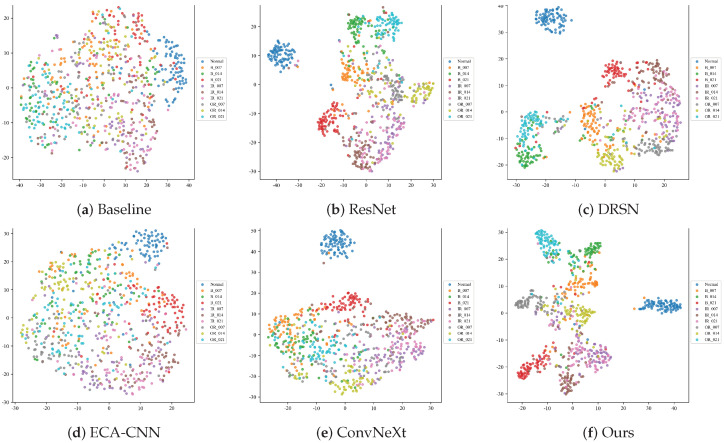
t-SNE feature manifolds on the CWRU dataset under Severe Noise (SNR = −10 dB).

To project the high-dimensional features into a 2D space and observe the clustering behavior in deeply corrupted environments, the t-Distributed Stochastic Neighbor Embedding (t-SNE) algorithm is utilized. Figure 8 illustrates the feature manifolds of the six models under the severe −10 dB noise condition.

When overwhelmed by drastic noise, models with weak feature extractors fail to establish distinct decision boundaries. As observed, the feature distributions of the Baseline and ECA-CNN models severely collapse. Different categories of defect samples are chaotically entangled in the feature space, exhibiting massive intra-class variance and almost zero inter-class distance, making correct classification practically impossible.

While deeper architectures such as ResNet, DRSN, and ConvNeXt attempt to aggregate features of the same class (e.g., the Normal state), they still suffer from severe overlapping at the cluster margins, particularly among complex defect categories like inner and outer race faults.

In stark contrast, the proposed MS-TCANet (Ours) exhibits the most discriminative feature manifold. Despite the −10 dB background noise, it successfully pulls samples of the same category tightly together (high intra-class compactness) while pushing different categories apart (high inter-class separability). This visualization perfectly echoes the quantitative results and confirms that the MS-TCANet possesses an exceptional ability to disentangle robust fault signatures from heavily masked signal distributions. 

### 4.3. Evaluation on PU Dataset

To further assess the diagnostic robustness, we evaluate the proposed MS-TCANet on the PU dataset, which is widely recognized for its diverse artificial and real damage patterns. Unlike the previous datasets, the PU dataset covers a broader range of bearing health conditions and fault severity levels, imposing greater demands on the feature learning capability of the diagnostic model under non-stationary interference. The diagnostic performance metrics under strong (−4 dB) and severe (−10 dB) noise conditions are detailed in Table 5.

Experimental results on the PU dataset systematically validate the superiority of the proposed MS-TCANet. Given the wide variety of real-world fatigue states and complex transmission paths in the PU dataset, diagnostic accuracy is generally suppressed across all models compared to lab-simulated datasets. However, under the −10 dB severe noise scenario, MS-TCANet consistently achieves the highest overall Accuracy (40.75%), Recall (40.75%), and F1-Score (40.86%), successfully outperforming standard residual networks and recent advanced architectures like ConvNeXt-TFA.

Crucially, MS-TCANet not only achieves the highest diagnostic metrics but also exhibits exceptional statistical stability. Under catastrophic −10 dB interference, our model effectively suppresses performance fluctuations, maintaining a remarkably low standard deviation (±0.56% on Accuracy). This explicitly proves that the proposed Peak-Aware Coordinate Attention mechanism successfully penetrates real-world severe noise floors to capture intrinsic physical fault signatures, guaranteeing a highly robust and reliable diagnostic performance in actual industrial deployments.

#### 4.3.1. Performance Under Extreme Scenarios (PU Dataset)

**Figure 9:**
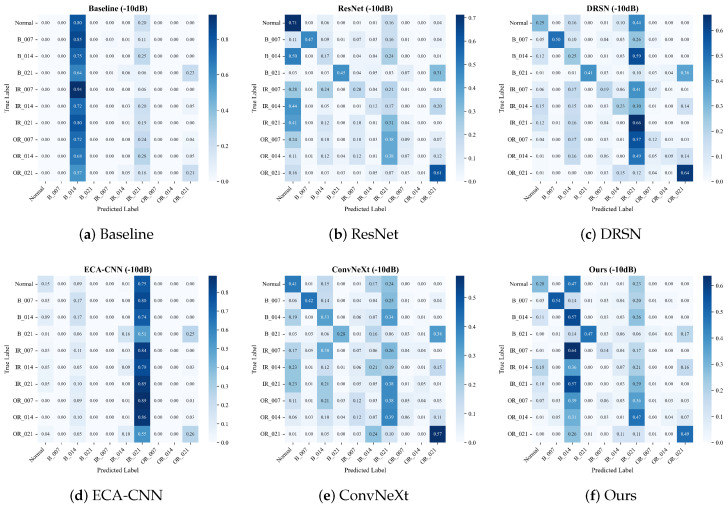
Confusion matrices on the PU dataset under Severe Noise (SNR = −10 dB).

To evaluate the ultimate robustness of the diagnostic networks, we further present the confusion matrices on the PU dataset under the demanding −10 dB noise condition (Figure 9). The PU dataset is characterized by real physical damage and complex operational dynamics, making it significantly more challenging than laboratory-simulated environments.

As illustrated in the matrices, the combination of real-world defects and severe noise triggers a severe “stress test” for all models, exposing their feature extraction bottlenecks. Under such catastrophic interference, comparative methods exhibit devastating mode collapse. Specifically, the Baseline model completely loses its discriminative boundaries, indiscriminately classifying the vast majority of samples (up to 94%) into a single B_014 category. Similarly, the recent ECA-CNN architecture suffers from a complete functional breakdown, collapsing its predictions overwhelmingly into the IR_021 mode (up to 89%).

While deeper architectures like ResNet and ConvNeXt resist total mode collapse, their diagnostic confidence is heavily diluted. Their predictions are widely scattered across off-diagonal regions, leading to severe inter-class confusion, particularly misclassifying various faults as the Normal or IR_021 states.

Distinguishing itself from conventional approaches, the proposed MS-TCANet (Ours) maintains the most coherent and distinct block-diagonal topology among all competitors. Despite the inevitable drop in absolute accuracy under the −10 dB floor, MS-TCANet robustly anchors the diagnostic boundaries, successfully preserving concentrated confidence on the diagonal for critical classes such as B_007 (54%), B_014 (57%), B_021 (47%), and OR_021 (49%). This visual evidence forcefully corroborates that the MS-TCANet acts as a robust “safety net” in harsh industrial environments, preventing the network from degenerating into a random guesser and significantly outperforming current state-of-the-art architectures.

#### 4.3.2. Attention Mechanism Under Real-World Damages

**Figure 10:**
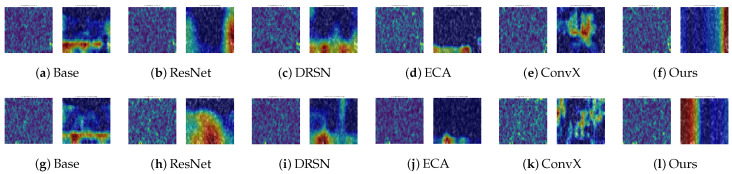
Grad-CAM heatmaps for B_007 (top) and OR_021 (bottom) on PU dataset at −4 dB SNR. The colors ranging from dark blue to dark red indicate the transition of attention weights from low to high.

Beyond quantitative metrics, it is vital to inspect whether the models genuinely capture fault signatures under real-world physical damages. Thus, Grad-CAM heatmaps are visualized on the PU dataset at the severe −10 dB SNR. We specifically select B_007 and OR_021 as the representative challenging categories to conduct a comprehensive visual comparison across all six models (Figure 10).

Unlike the artificially induced defects in the CWRU dataset, the natural wear and tear in the PU dataset produce much weaker impact energy, which is easily buried by the −10 dB background noise. Consequently, models lacking robust feature selection mechanisms completely lose their focus. As observed in Figure 10, the attention maps of the Baseline and ECA-CNN models are heavily fragmented. They are erroneously activated by random global noise patches rather than actual fault signatures, directly explaining their severe mode collapse in the confusion matrices.

Furthermore, although deeper architectures like ResNet, DRSN, and ConvNeXt attempt to capture structural features from the heavily corrupted STFT spectrum, their focal regions remain dispersed and fail to form continuous frequency bands.

Conversely, the proposed MS-TCANet exhibits exceptional noise immunity. Its attention heatmaps present highly concentrated and continuous vertical bands. These highlighted regions perfectly align with the resonance frequency bands excited by the periodic transient impulses of the bearing defects in the time-frequency domain. This compelling visual evidence forcefully corroborates that the proposed multi-scale peak-aware mechanism can effectively penetrate real-world severe noise floors to capture intrinsic physical fault signatures, thereby guaranteeing robust diagnostic performance.

#### 4.3.3. Feature Manifold Visualization Under Real-World Damages

**Figure 11:**
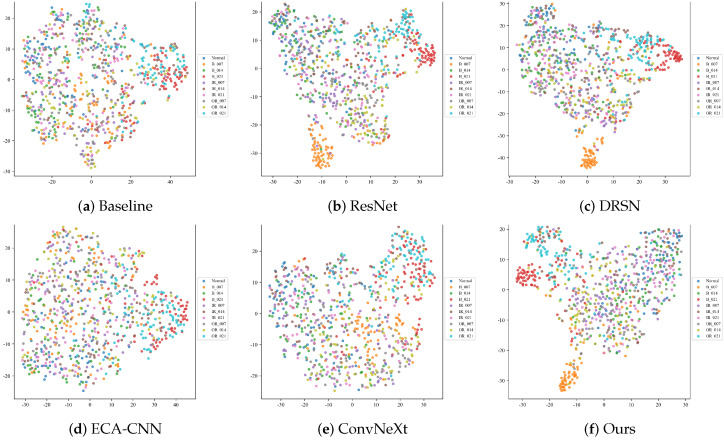
t-SNE feature manifolds on the PU dataset under Severe Noise (SNR = −10 dB).

To explicitly uncover the underlying reasons for the drastic mode collapse observed in comparative models, we employ the t-SNE algorithm to visualize the high-dimensional feature distributions on the PU dataset under the severe −10 dB noise condition (Figure 11). The combination of real-world physical damage and severe background interference makes feature clustering exceptionally difficult.

As clearly demonstrated, models lacking adaptive feature extraction capabilities experience complete feature space degradation. The feature manifolds of the Baseline and ECA-CNN models are entirely chaotic, with samples from all categories randomly scattered. There is virtually zero intra-class compactness or inter-class separability, which fundamentally explains their functional breakdown and mode collapse in the classification phase.

Similarly, while complex network structures such as ResNet, DRSN, and ConvNeXt attempt to group certain distinct faults (e.g., partially isolating the orange cluster for B_007 and the red cluster for B_021), they fundamentally fail to disentangle the massive overlapping manifolds of the remaining categories in the central region. The boundaries between most defect types are highly blurred, indicating that these conventional networks fail to extract robust discriminative signatures from the corrupted physical signals. 

Overcoming these limitations, the proposed MS-TCANet (Ours) exhibits an outstanding capability to defend the feature manifold against severe noise. Despite the highly challenging conditions, MS-TCANet successfully pulls samples of the same fault category together. It not only achieves highly cohesive, well-separated clusters for specific classes (e.g., the distinct orange cluster at the bottom and the red cluster on the left) but also significantly reduces the chaotic overlap in the center compared to all competitors. This visualization perfectly corroborates our previous quantitative findings: the multi-scale peak-aware mechanisms empower MS-TCANet to persistently disentangle robust physical fault signatures from deeply masked industrial signals, thereby ensuring superior diagnostic separability.

### 4.4. Evaluation on XJTU-SY Dataset

The XJTU-SY dataset, consisting of vibration signals collected from bearings under different operating conditions, serves as a more challenging benchmark due to its non-stationary and varying operating parameters. We apply the same evaluation protocol to verify the robustness of MS-TCANet against the combined challenges of varying working conditions and high-intensity ambient noise. The diagnostic performance metrics under strong (−4 dB) and severe (−10 dB) noise conditions are detailed in Table 6.

The XJTU-SY results demonstrate that MS-TCANet maintains superior diagnostic capabilities even in the presence of varying operating conditions. In the −10 dB severe noise scenario, the MS-TCANet achieves an accuracy of 46.33%, representing a notable improvement over ResNet (44.17%) and DRSN (43.62%). This confirms that the proposed dual-pooling strategy is capable of suppressing the non-stationary components of noise while retaining critical impulsive features required for fault identification.

#### 4.4.1. Diagnostic Consistency Under Time-Varying Conditions (XJTU-SY)

**Figure 12:**
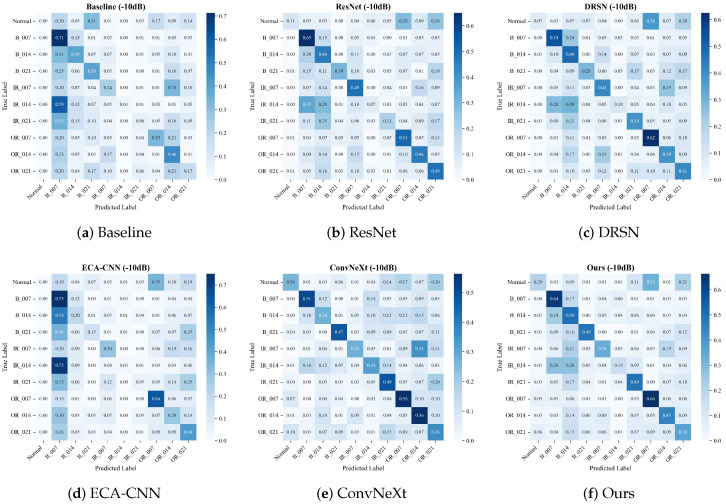
Confusion matrices on the XJTU-SY dataset under Severe Noise (SNR = −10 dB).

To further validate the generalization and robustness of the proposed method under varying operating conditions, we evaluate the models on the XJTU-SY dataset at −10 dB SNR. Unlike previous datasets, the XJTU-SY dataset captures the full run-to-failure degradation process, introducing significant temporal distribution shifts that severely challenge the models’ diagnostic consistency. The corresponding confusion matrices are shown in Figure 12.

When the time-varying fault signatures are deeply buried by severe −10 dB noise, most comparative models suffer from severe decision boundary blurring. For instance, the Baseline model completely fails to identify the Normal state (yielding 0% accuracy) and scatters its predictions chaotically. Alarmingly, the ECA-CNN model suffers from another severe mode collapse, misclassifying the vast majority of samples into the OR_007 category regardless of their true labels. Even deep architectures like ConvNeXt and ResNet struggle to maintain confidence, exhibiting highly diluted diagonals and severe off-diagonal confusion.

On the contrary, our multi-scale peak-aware design empowers the MS-TCANet (Ours) to successfully withstand this extreme limit-testing scenario. It is the only model that consistently preserves a distinct block-diagonal topology. Notably, MS-TCANet achieves impressive accuracies of 64% on the B_007 class and 66% on the OR_007 class, significantly outperforming all competitors. This evidence firmly establishes that the multi-scale peak-aware design can effectively extract invariant physical fault signatures, ensuring highly reliable diagnostics even when operating under the dual threats of time-varying conditions and severe environmental noise.

#### 4.4.2. Attention Mechanism Under Time-Varying Degradation

**Figure 13:**
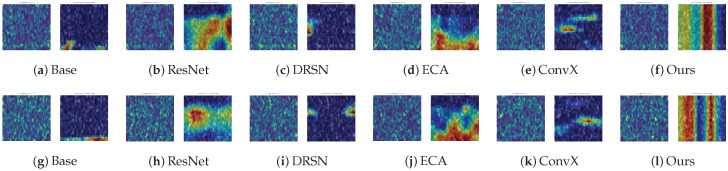
Grad-CAM heatmaps for B_007 (top) and OR_007 (bottom) on XJTU-SY dataset at −4 dB SNR. The colors ranging from dark blue to dark red indicate the transition of attention weights from low to high.

To explicitly demystify how the models cope with the dual threats of time-varying degradation and severe environmental noise, we generate the attention heatmaps for the XJTU-SY dataset at −10 dB SNR. We select the challenging B_007 and OR_007 categories for a comprehensive visualization across the six comparative architectures (Figure 13).

Because the XJTU-SY dataset records the full run-to-failure lifecycle, the fault impulse energy inherently fluctuates and is often extremely weak, especially when heavily masked by −10 dB background noise. Consequently, the original STFT spectrum is almost entirely corrupted by random high-frequency interference. Under such conditions, models lacking temporal consistency and adaptive feature selection suffer from severe attention dispersion. For instance, the attention mechanisms of the Baseline and ECA-CNN models are erroneously trapped by chaotic noise patches, completely missing the intrinsic defect frequencies. This visual evidence fundamentally explains their severe prediction scattering and mode collapse observed in the classification phase.

Although advanced networks such as ConvNeXt and ResNet attempt to trace the fault signatures, their attention distributions remain fragmented and easily interrupted by severe noise bursts along the temporal axis.

Remarkably, the proposed MS-TCANet acts as a robust feature tracker. Despite the severe SNR drop and the temporal distribution shifts inherent to the run-to-failure data, its attention heatmaps consistently highlight highly concentrated and continuous vertical bands. These distinct bands indicate that the network precisely locks onto the resonance frequency excited by the bearing defects across the entire time window. This compelling visualization demonstrates that the proposed multi-scale peak-aware mechanism can robustly filter out non-stationary background noise and capture invariant physical fault signatures throughout the degradation process.

#### 4.4.3. Feature Manifold Visualization Under Time-Varying Conditions

**Figure 14:**
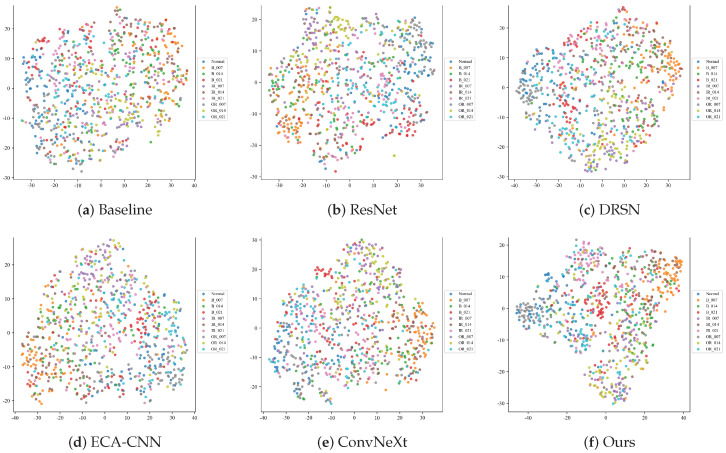
t-SNE feature manifolds on the XJTU-SY dataset under Severe Noise (SNR = −10 dB).

The dual challenges of full lifecycle degradation and severe background noise make feature clustering exceptionally difficult. Figure 14 visualizes the t-SNE projections to reveal how different models handle such temporal shifts at −10 dB SNR.

As expected, the time-varying nature of the fault impulses, compounded by heavy noise, leads to severe feature entanglement for conventional models. The feature spaces of the Baseline and ECA-CNN architectures are completely chaotic, with samples from different health states severely overlapping. This lack of discriminative boundaries directly accounts for their severe mode collapse and poor diagnostic performance. Furthermore, while deeper networks like ConvNeXt and DRSN successfully isolate the Normal state (the blue cluster), they fail to disentangle the complex fault categories, resulting in massive inter-class confusion among the defect samples.

Demonstrating superior resilience, the proposed MS-TCANet (Ours) exhibits an exceptional ability to maintain a highly discriminative feature manifold. Despite the severe −10 dB interference and the temporal shifts inherent to the run-to-failure data, MS-TCANet successfully pulls samples of the same category tightly together while pushing different categories apart. Most fault categories form distinct, well-separated clusters with high intra-class compactness. This compelling visualization demonstrates that the multi-scale peak-aware mechanism acts as a robust feature extractor, capable of discovering invariant and reliable diagnostic signatures even in the most demanding dynamic environments.

### 4.5. Model Complexity and Efficiency Analysis

For actual industrial deployment in condition-based maintenance (CBM) systems, deep learning models must balance diagnostic accuracy with computational efficiency. To evaluate the feasibility of deploying MS-TCANet on edge sensing devices, we analyzed the model complexity using three metrics: Parameter volume (Params), Floating Point Operations (FLOPs), and Inference Time per sample. The inference time is averaged over 1000 iterations using a batch size of 1 on an NVIDIA RTX 2060 GPU to simulate real-time single-sample monitoring. As shown in Table 7, highly simplified baseline networks possess extremely low parameter counts (e.g., 0.02 M for the Baseline 2DCNN) and fast inference speeds. However, as demonstrated in previous experiments, these simplistic architectures suffer from severe mode collapse under heavy noise conditions. The proposed MS-TCANet introduces asymmetric multi-scale large kernels and the dual-pooling PACA mechanism to extract robust features from noisy signals, which naturally results in a slight increase in complexity. Despite this, MS-TCANet remains compact at 1.63 M parameters and 0.12 G FLOPs. Its single-sample inference time is 11.69 ms, allowing it to process approximately 85 samples per second. This fulfills the sub-second latency requirements for real-time industrial deployment. Trading a fractional increase in computation time for a substantial improvement in diagnostic reliability under extreme noise (e.g., −10 dB SNR) represents a practical trade-off for industrial applications.

Additional qualitative results (confusion matrices, attention heatmaps, and t-SNE visualizations) under the strong noise condition (−4 dB) for all three datasets are provided in Appendix A.

## 5. Ablation Study and Mechanism Analysis

To quantitatively evaluate the individual contribution and indispensability of each key component in the proposed MS-TCANet, a rigorous ablation study was conducted. We constructed three ablated variants by progressively removing core modules from the full architecture:

1. No Multi-Scale: The 7×7 large-kernel convolution branch is removed, retaining only the 3×3 local extraction path.
2. No PACA: The Peak-Aware Coordinate Attention (PACA) module is completely removed.
3. No Max-Pooling: The parallel max-pooling branch in PACA is disabled, degrading it to a standard average-pooling-based coordinate attention mechanism.
4. Full Model (Ours): The proposed MS-TCANet with all components enabled.

### 5.1. Performance Under Severe Noise Conditions

Table 8 summarizes the diagnostic accuracy of the ablated variants on the XJTU-SY dataset under severe noise regimes. As shown in the results, removing any component leads to a noticeable performance decline. Specifically, the removal of the PACA module results in the most significant drop at −10 dB, highlighting its critical role in noise immunity. Furthermore, comparing the “Full Model” with the “No Max-Pooling” variant explicitly proves that the peak-aware branch is the key differentiator for preserving diagnostic evidence from heavy background interference.

Moreover, the ablation results highlight a synergistic relationship between the multi-scale convolution block and the PACA module. The multi-scale design acts as a feature generator, utilizing 3×3 and 7×7 kernels to capture both localized short-duration impacts and broader long-period degradation modulations. However, generating rich multi-scale representations is insufficient if the subsequent attention aggregation dilutes the impulse energy. PACA serves as an essential feature selector that complements the multi-scale block. Without PACA, the extracted multi-scale features suffer from over-smoothing in noisy environments. Conversely, without the multi-scale block, PACA is restricted to local peak detection and misses periodic structural trends. This synergy enables the full MS-TCANet to outperform its individual sub-variants.

### 5.2. Mechanism Discussion: Why Peak-Awareness Matters

The effectiveness of MS-TCANet, particularly the PACA mechanism, can be interpreted through physical signal characteristics. Localized bearing defects manifest as sharp transient impulses. Conventional attention modules, which rely solely on Global Average Pooling (GAP), essentially act as low-pass filters. While GAP stabilizes the global representation, it fatally dilutes the energy of high-frequency, low-duty-cycle transient peaks under severe noise.

By introducing the parallel max-pooling branch, PACA acts mathematically as a non-linear peak detector. Physically, early bearing defects generate sparse, high-amplitude transient impulses. While average pooling smooths the global representation, max pooling explicitly captures the maximum amplitude responses corresponding to these transient impacts along the spatial dimensions. This ensures that the network preserves informative diagnostic signatures—specifically the sharp physical impact peaks—instead of allowing them to be diluted by the low-pass filtering effect of standard attention modules. This synergetic effect between global stability (average pooling) and impulse sensitivity (max pooling) enables MS-TCANet to maintain reliable diagnostic capabilities even in noisy environments.

## 6. Conclusions

In this paper, we propose the MS-TCANet to address the challenge of bearing fault diagnosis under severe noise interference in industrial environments. To mitigate the feature over-smoothing issue caused by conventional global average pooling, we designed a Peak-Aware Coordinate Attention (PACA) mechanism. By integrating directional max-pooling with average-pooling, the model captures sparse transient impact peaks while maintaining global feature stability. Coupled with a multi-scale large-kernel convolution block, the network effectively extracts both short-duration impulses and long-period degradation modulations. Rather than merely pursuing absolute accuracy metrics, evaluations across three datasets with varying physical difficulties demonstrate that the proposed architecture provides more stable feature decision boundaries than conventional models, particularly under the demanding −10 dB SNR condition.

Despite the demonstrated robustness, this study primarily evaluated the model using AWGN as a standard benchmark stress test. The explicit modeling of real non-Gaussian industrial interferences, such as transient fluctuations and spikes correlated with PWM inverter drives, severe mechanical impulse noise, and transmission path attenuation, remains a limitation. Future research will explore specialized noise-reduction modules tailored for these specific non-Gaussian noise distributions and investigate model compression techniques to facilitate low-latency deployment on resource-constrained edge sensing devices.

## Figures and Tables

**Table 1:** Description of the 10 health states in the CWRU dataset.

Health State	Fault Diameter (Inch)	Fault Location	Class Label
Normal	0.000	N/A	0
Ball Defect	0.007	Rolling Element	B_007 (1)
	0.014	Rolling Element	B_014 (2)
	0.021	Rolling Element	B_021 (3)
Inner Race Defect	0.007	Inner Race	IR_007 (4)
	0.014	Inner Race	IR_014 (5)
	0.021	Inner Race	IR_021 (6)
Outer Race Defect	0.007	Outer Race (Orthogonal)	OR_007 (7)
	0.014	Outer Race (Orthogonal)	OR_014 (8)
	0.021	Outer Race (Orthogonal)	OR_021 (9)

**Table 2:** Description of the selected 10 health states mapped from the PU dataset.

Health State	Damage Type	Fault Location	Unified Class Label
Normal	Undamaged	N/A	0
Ball Defect	Severity Level 1	Rolling Element	B_007 (1)
	Severity Level 2	Rolling Element	B_014 (2)
	Severity Level 3	Rolling Element	B_021 (3)
Inner Race Defect	Severity Level 1	Inner Race	IR_007 (4)
	Severity Level 2	Inner Race	IR_014 (5)
	Severity Level 3	Inner Race	IR_021 (6)
Outer Race Defect	Severity Level 1	Outer Race	OR_007 (7)
	Severity Level 2	Outer Race	OR_014 (8)
	Severity Level 3	Outer Race	OR_021 (9)

**Table 3:** Description of the unified 10 health states mapped from the XJTU-SY run-to-failure degradation stages.

Health State	Degradation Stage	Fault Location	Unified Class Label
Normal	Healthy Operation	N/A	0
Ball Defect	Early Stage (Incipient)	Rolling Element	B_007 (1)
	Middle Stage (Moderate)	Rolling Element	B_014 (2)
	Late Stage (Severe)	Rolling Element	B_021 (3)
Inner Race Defect	Early Stage (Incipient)	Inner Race	IR_007 (4)
	Middle Stage (Moderate)	Inner Race	IR_014 (5)
	Late Stage (Severe)	Inner Race	IR_021 (6)
Outer Race Defect	Early Stage (Incipient)	Outer Race	OR_007 (7)
	Middle Stage (Moderate)	Outer Race	OR_014 (8)
	Late Stage (Severe)	Outer Race	OR_021 (9)

**Table 4:** Performance metrics on CWRU dataset under −4 dB and −10 dB noise conditions.

Model	−4 dB (Strong Noise)			−10 dB (Severe Noise)		
	Acc (%)	Rec (%)	F1 (%)	Acc (%)	Rec (%)	F1 (%)
Baseline_2DCNN	92.31 ± 0.49	92.20 ± 0.50	92.16 ± 0.59	46.97 ± 1.46	46.54 ± 1.50	46.14 ± 1.41
ECA-CNN	94.89 ± 0.54	94.82 ± 0.54	94.81 ± 0.55	57.75 ± 1.70	57.42 ± 1.70	56.78 ± 2.05
ConvNeXt-TFA	94.97 ± 0.59	94.90 ± 0.60	94.92 ± 0.59	64.78 ± 1.30	64.41 ± 1.32	64.23 ± 1.18
ResNet	95.14 ± 0.12	95.07 ± 0.13	95.08 ± 0.12	69.47 ± 0.55	69.13 ± 0.56	68.90 ± 0.74
DRSN	95.75 ± 0.77	95.69 ± 0.78	95.70 ± 0.77	70.22 ± 1.34	69.87 ± 1.36	69.69 ± 1.46
Ours (MS-TCANet)	96.36 ± 0.18	96.31 ± 0.19	96.32 ± 0.18	74.67 ± 1.24	74.37 ± 1.26	74.36 ± 1.28

**Table 5:** Performance metrics on PU dataset under −4 dB and −10 dB noise conditions.

Model	−4 dB (Strong Noise)			−10 dB (Severe Noise)		
	Acc (%)	Rec (%)	F1 (%)	Acc (%)	Rec (%)	F1 (%)
Baseline_2DCNN	57.30 ± 0.99	57.30 ± 0.99	56.05 ± 1.01	28.07 ± 1.14	28.07 ± 1.14	27.89 ± 1.13
ECA-CNN	60.42 ± 0.65	60.42 ± 0.65	59.52 ± 0.52	29.93 ± 1.87	29.93 ± 1.87	30.70 ± 1.63
ConvNeXt-TFA	65.20 ± 1.45	65.20 ± 1.45	64.73 ± 1.45	34.77 ± 0.79	34.77 ± 0.79	34.66 ± 0.87
DRSN	67.90 ± 0.73	67.90 ± 0.73	67.22 ± 0.68	40.05 ± 0.88	40.05 ± 0.88	40.44 ± 0.73
ResNet	68.03 ± 0.64	68.03 ± 0.64	67.28 ± 0.93	39.73 ± 1.46	39.73 ± 1.46	39.81 ± 1.26
Ours (MS-TCANet)	69.70 ± 0.94	69.70 ± 0.94	69.28 ± 0.80	40.75 ± 0.56	40.75 ± 0.56	40.86 ± 0.78

**Table 6:** Performance metrics on XJTU-SY dataset under −4 dB and −10 dB noise conditions.

Model	−4 dB (Strong Noise)			−10 dB (Severe Noise)		
	Acc (%)	Rec (%)	F1 (%)	Acc (%)	Rec (%)	F1 (%)
Baseline_2DCNN	80.98 ± 0.99	80.98 ± 0.99	80.98 ± 0.98	31.62 ± 1.54	31.62 ± 1.54	30.48 ± 2.04
ConvNeXt-TFA	89.20 ± 1.09	89.20 ± 1.09	89.28 ± 1.06	43.17 ± 1.16	43.17 ± 1.16	42.90 ± 1.42
DRSN	91.58 ± 1.47	91.58 ± 1.47	91.61 ± 1.45	43.62 ± 1.16	43.62 ± 1.16	43.07 ± 1.35
ECA-CNN	87.05 ± 1.02	87.05 ± 1.02	87.10 ± 0.99	35.85 ± 1.65	35.85 ± 1.65	35.23 ± 1.96
ResNet	89.60 ± 2.05	89.60 ± 2.05	89.58 ± 2.15	44.17 ± 0.48	44.17 ± 0.48	43.72 ± 0.80
Ours (MS-TCANet)	93.80 ± 0.17	93.80 ± 0.17	93.83 ± 0.17	46.33 ± 0.71	46.33 ± 0.71	46.05 ± 0.88

**Table 7:** Comparison of Model Complexity and Inference Efficiency.

Model	Parameters (M)	FLOPs (G)	Inference Time (ms/Sample)
Baseline_2DCNN	0.02	0.01	0.92
ConvNeXt-TFA	0.07	0.01	1.06
ResNet	0.28	0.01	1.28
ECA-CNN	0.09	0.01	1.41
DRSN	0.33	0.02	2.68
Ours (MS-TCANet)	1.63	0.12	11.69

**Table 8:** Comprehensive performance comparison of ablation variants (XJTU-SY Dataset).

Variant	SNR (dB)	Accuracy (%)	Recall (%)	F1-Score (%)
No Multi-Scale	−10	42.45 ± 1.34	42.45 ± 1.34	42.24 ± 1.35
No PACA	−10	38.00 ± 0.77	38.00 ± 0.77	37.78 ± 0.79
No Max-Pooling	−10	44.62 ± 1.01	44.62 ± 1.01	44.30 ± 1.02
Full Model	−10	46.33 ± 0.71	46.33 ± 0.71	46.05 ± 0.88

**Figure A1:**
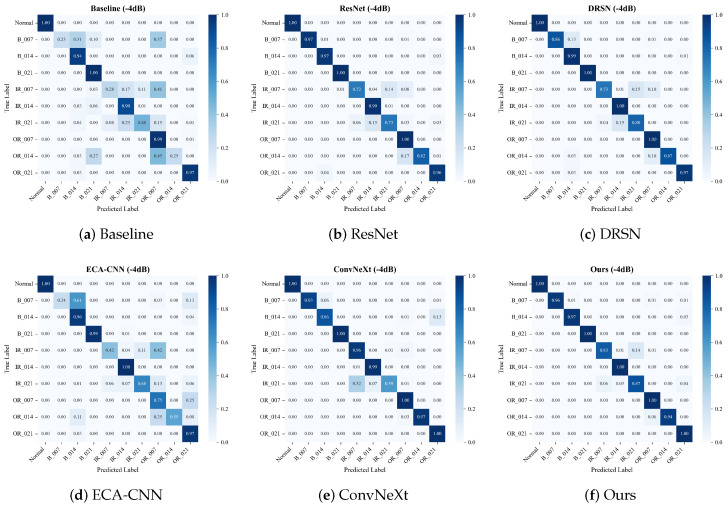
Confusion matrices on the CWRU dataset under strong noise (SNR = −4 dB).

**Figure A2:**
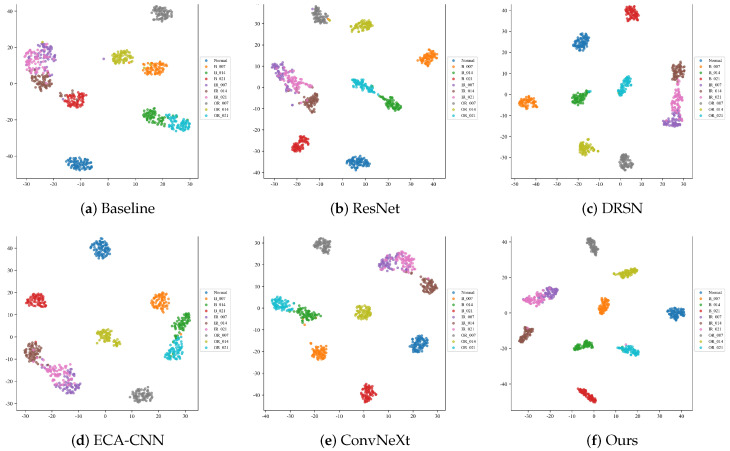
t-SNE feature manifolds on the CWRU dataset under strong noise (SNR = −4 dB).

**Figure A3:**
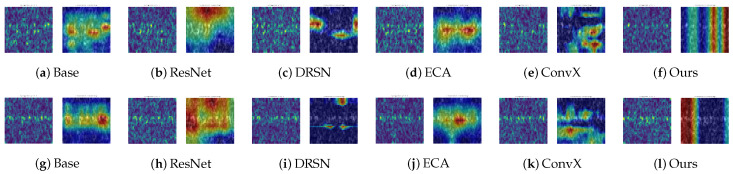
Attention heatmaps for B_007 (top) and IR_007 (bottom) on CWRU dataset at −4 dB SNR.

**Figure A4:**
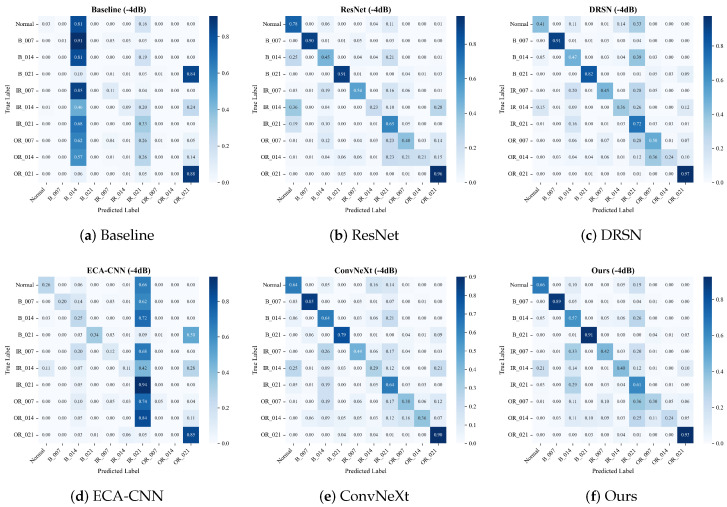
Confusion matrices on the PU dataset under strong noise (SNR = −4 dB).

**Figure A5:**
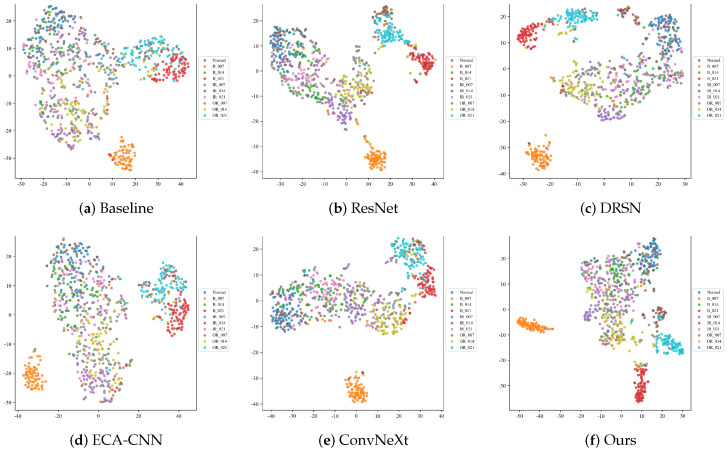
t-SNE feature manifolds on the PU dataset under strong noise (SNR = −4 dB).

**Figure A6:**
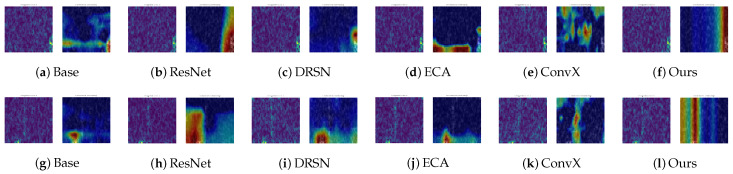
Attention heatmaps for B_007 (top) and OR_021 (bottom) on PU dataset at −4 dB SNR. The colors ranging from dark blue to dark red indicate the transition of attention weights from low to high.

**Figure A7:**
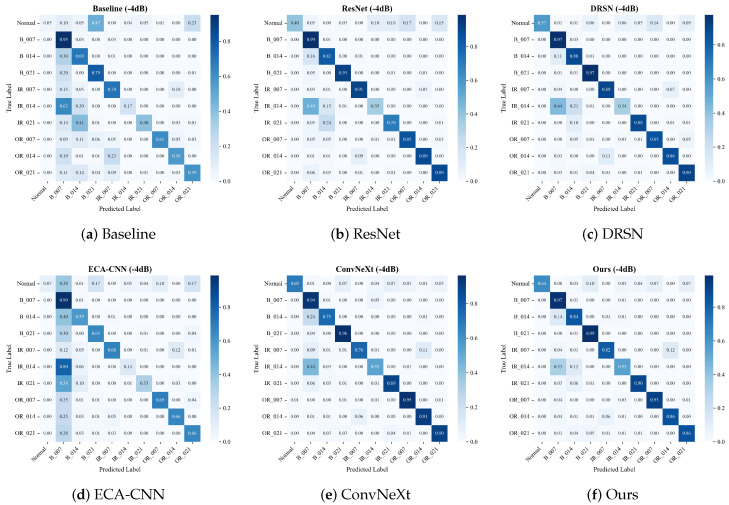
Confusion matrices on the XJTU-SY dataset under strong noise (SNR = −4 dB).

**Figure A8:**
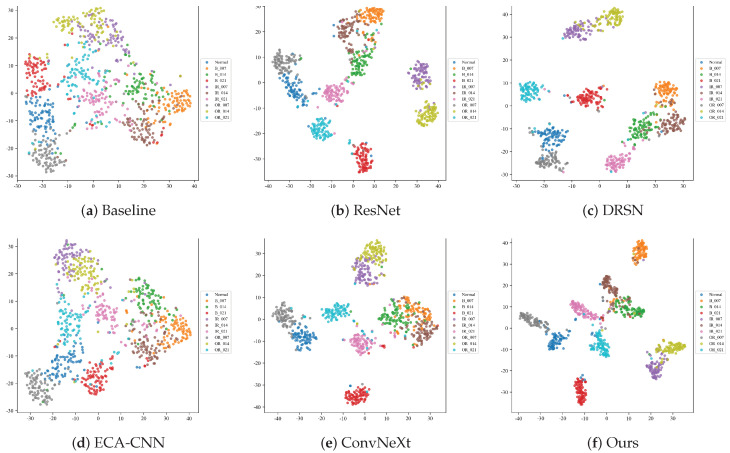
t-SNE feature manifolds on the XJTU-SY dataset under strong noise (SNR = −4 dB).

**Figure A9:**
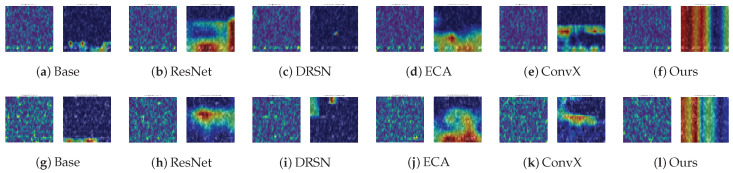
Attention heatmaps for B_007 (top) and OR_007 (bottom) on XJTU-SY dataset at −4 dB SNR. The colors ranging from dark blue to dark red indicate the transition of attention weights from low to high.

## Data Availability

The datasets (CWRU, PU, XJTU-SY) analyzed during the current study are available in the public domain.

## Appendix A. Supplementary Visualizations Under Strong Noise (−4 dB)

To provide a comprehensive evaluation of the diagnostic robustness, this appendix presents the qualitative visual results under the strong noise condition (SNR = −4 dB) for all three benchmark datasets. All results are presented using the same format as the main text.

## References

[1] Lei Y., Jia F., Lin J., Xing S., Ding S.X. (2020). Applications of machine learning to machine fault diagnosis: A review and roadmap. Mech. Syst. Signal Process..

[2] Zhao R., Yan R., Chen Z., Mao K., Wang P., Gao R.X. (2019). Deep learning and its applications to machine health monitoring. Mech. Syst. Signal Process..

[3] Hoang D.T., Kang H.J. (2019). A survey on deep learning based bearing fault diagnosis. Neurocomputing.

[4] Randall R.B., Antoni J. (2011). Rolling element bearing diagnostics—A tutorial. Mech. Syst. Signal Process..

[5] Liu R., Yang B., Zio M., Chen X. (2018). Artificial intelligence for fault diagnosis of rotating machinery: A review. Mech. Syst. Signal Process..

[6] Zhang W., Peng G., Li C., Chen Y., Zhang Z. (2017). A new deep learning model for fault diagnosis with good anti-noise and domain adaptation ability on raw vibration signals. Sensors.

[7] Jia F., Lei Y., Lin J., Zhou X., Lu N. (2016). Deep neural networks: A promising tool for fault characteristic mining and intelligent diagnosis of rotating machinery with massive data. Mech. Syst. Signal Process..

[8] Wang J., Ma Y., Zhang L., Gao R.X., Wu D. (2021). A comprehensive review of deep learning-based fault diagnosis of machinery. Mech. Syst. Signal Process..

[9] Venkatachalam Y., Subbaiyan T. (2025). Intelligent fault diagnosis in power systems: A comparative analysis of machine learning-based algorithms. Expert Syst. Appl..

[10] Wan Y., Wang S., Liu D. (2025). Fault diagnosis using liquid state machine with spiking-timing-dependent plasticity learning rule. Expert Syst. Appl..

[11] Jiang H., Li X., Shao H., Zhao K. (2018). A new fault diagnosis method for rotary machinery based on one-dimensional convolutional neural network. Sensors.

[12] Zhao Z., Li T., Wu J., Sun C., Wang S., Yan R., Chen X. (2020). Deep learning algorithms for rotating machinery intelligent diagnosis: An open source benchmark study. ISA Trans..

[13] He K., Zhang X., Ren S., Sun J. (2016). Deep residual learning for image recognition. Proceedings of the IEEE Conference on Computer Vision and Pattern Recognition.

[14] Zhao M., Zhong S., Fu X., Tang B., Dong S., Pecht M. (2018). Deep residual networks with dynamically weighted wavelet coefficients for fault diagnosis of planetary gearboxes. IEEE Trans. Ind. Electron..

[15] Zhao M., Zhong S., Fu X., Tang B., Dong S., Pecht M. (2019). Deep residual shrinkage networks for fault diagnosis. IEEE Trans. Ind. Inform..

[16] Zhao M., Kang M., Tang B., Pecht M. (2019). Multiple wavelet coefficients fusion in deep residual networks for fault diagnosis. IEEE Trans. Ind. Electron..

[17] Zhang W., Li C., Peng G., Chen Y., Zhang Z. (2018). A deep convolutional neural network with new training methods for bearing fault diagnosis under noisy environment and different working load. Mech. Syst. Signal Process..

[18] Li X., Zhang W., Ding Q. (2020). Domain adaptation in bearing fault diagnosis: A review. IEEE Trans. Instrum. Meas..

[19] Zou Y., Luo S., Wu X., Jiang Q., Zhang P., Du T., Zhang Y., Sun P., Xu M. (2026). MEHD-Net: A multimodal-enhanced hybrid denoising network for marine gearbox bearing fault diagnosis under extreme noise. Measurement.

[20] Shi Y., Wang F., Huang Y. (2025). An adaptive cyclostationarity feature mode decomposition for rolling bearing fault diagnosis under strong background noise. Measurement.

[21] WEI W., Yuan Y. (2026). VMD-DCA-BiGRU wind turbine bearing fault diagnosis method with attention mechanism integration. Measurement.

[22] Li X., Zhang W., Ding Q., Sun J.Q. (2019). Multi-layer domain adaptation method for rolling bearing fault diagnosis. Signal Process..

[23] Han T., Liu C., Yang W., Jiang D. (2020). Deep transfer network with joint distribution adaptation: A new intelligent fault diagnosis framework for industry application. ISA Trans..

[24] Shao H., Jiang H., Zhao H., Wang F. (2017). A novel deep autoencoder feature learning method for rotating machinery fault diagnosis. Mech. Syst. Signal Process..

[25] Lv H., Chen J., Pan T., Zhang T., Liao Y., Liu J. (2022). Attention mechanism in intelligent fault diagnosis of machinery: A review of technique and application. Measurement.

[26] Hu J., Shen L., Sun G. (2018). Squeeze-and-excitation networks. Proceedings of the IEEE Conference on Computer Vision and Pattern Recognition.

[27] Woo S., Park J., Lee J.Y., Kweon I.S. (2018). Cbam: Convolutional block attention module. Proceedings of the European Conference on Computer Vision (ECCV).

[28] Wang Q., Wu B., Zhu P., Li P., Zuo W., Hu Q. (2020). ECA-Net: Efficient channel attention for deep convolutional neural networks. Proceedings of the IEEE/CVF Conference on Computer Vision and Pattern Recognition.

[29] Hou Q., Zhou D., Feng J. (2021). Coordinate attention for efficient mobile network design. Proceedings of the IEEE/CVF Conference on Computer Vision and Pattern Recognition.

[30] Xiao Z., Xu Y., Cui J. (2025). VFQB: A Novel Deep Learning Model for Rolling Bearing Fault Diagnosis. Sensors.

[31] Guo H., Ping D., Wang L., Zhang W., Wu J., Ma X., Xu Q., Lu Z. (2025). Fault Diagnosis Method of Rolling Bearing Based on 1D Multi-Channel Improved Convolutional Neural Network in Noisy Environment. Sensors.

[32] Kim Y., Kim Y.K. (2023). Time-Frequency Multi-Domain 1D Convolutional Neural Network with Channel-Spatial Attention for Noise-Robust Bearing Fault Diagnosis. Sensors.

[33] He J., Wu P., Tong Y., Zhang X., Lei M., Gao J. (2021). Bearing Fault Diagnosis via Improved One-Dimensional Multi-Scale Dilated CNN. Sensors.

[34] Gao J., Pan R., Yuan Y., Zhou J., Ding H., Kong W. (2026). A novel multiaxial fatigue life prediction method based on multi-scale physical neural network. Reliab. Eng. Syst. Saf..

[35] Lin M., Chen Q., Yan S. (2013). Network in network. arXiv.

[36] Wang Y., Ding X., Zeng Q., Wang L., Shao Y. (2021). Intelligent rolling bearing fault diagnosis via vision ConvNet. IEEE Sens. J..

[37] Liu Z., Mao H., Wu C.Y., Feichtenhofer C., Darrell T., Xie S. (2022). A convnet for the 2020s. Proceedings of the IEEE/CVF Conference on Computer Vision and Pattern Recognition.

[38] Ding X., Zhang X., Ma Y., Han J., Ding G., Sun J. (2022). Scaling up your kernels to 31x31: Revisiting large kernel design in cnns. Proceedings of the IEEE/CVF Conference on Computer Vision and Pattern Recognition.

[39] Smith W.A., Randall R.B. (2015). Rolling element bearing diagnostics using the Case Western Reserve University data: A benchmark study. Mech. Syst. Signal Process..

[40] Lessmeier C., Kimotho J.K., Zimmer D., Sexton W. Condition monitoring of bearing damage in electromechanical drive systems by using motor current signals of electric motors: A benchmark data set for data-driven classification. Proceedings of the European Conference of the Prognostics and Health Management Society.

[41] Wang B., Lei Y., Li N., Li N. (2018). A hybrid prognostics approach for estimating remaining useful life of rolling element bearings. IEEE Trans. Reliab..

